# Inlet protein aggregation: a new mechanism for lubricating film formation with model synovial fluids

**DOI:** 10.1177/0954411911401306

**Published:** 2011-07

**Authors:** J Fan, C W Myant, R Underwood, P M Cann, A Hart

**Affiliations:** 1 Tribology Group, Department of Mechanical Engineering, Imperial College London, London, UK; 2 London Retrieval Centre, Charing Cross Hospital, Imperial College London, London, UK

**Keywords:** artificial hip joint, synovial fluid, boundary lubrication, CoCrMo alloy

## Abstract

This paper reports a fundamental study of lubricant film formation with model synovial fluid components (proteins) and bovine serum (BS). The objective was to investigate the role of proteins in the lubrication process. Film thickness was measured by optical interferometry in a ball-on-disc device (mean speed range of 2–60 mm/s). A commercial cobalt–chromium (CoCrMo) metal femoral head was used as the stationary component. The results for BS showed complex time-dependent behaviour, which was not representative of a simple fluid. After a few minutes sliding BS formed a thin adherent film of 10–20 nm, which was attributed to protein absorbance at the surface. This layer was augmented by a hydrodynamic film, which often increased at slow speeds. At the end of the test deposited surface layers of 20–50 nm were measured. Imaging of the contact showed that at slow speeds an apparent ‘phase boundary’ formed in the inlet just in front of the Hertzian zone. This was associated with the formation of a reservoir of high-viscosity material that periodically moved through the contact forming a much thicker film. The study shows that proteins play an important role in the film-forming process and current lubrication models do not capture these mechanisms.

## 1 INTRODUCTION

Despite significant advances in recent years, surface wear of artificial hip implants remains a serious concern. For metal-on-polymer (MOP) joints the formation of polyethylene wear particles can lead to osteolysis and bone loss resulting in loosening of the implant [**1**]. As a result some surgeons advise that these joints are not employed for younger or more active patients. Thus to increase implant longevity second-generation metal-on-metal (MOM) joints have been employed. Although these give much lower surface wear they do result in the formation of metallic nanoparticles, which give increased metal ion levels in the blood [**2**] and are associated with periprosthetic lesions [**3**]. Clearly the reduction of wear is still an important goal for tribological research. If new low-wear materials and surface coatings for artificial joints are to be developed, it is important to understand the fundamental lubrication mechanisms and the influence of synovial fluid (SF) chemistry. The present study explores the role of SF proteins.

Implant wear is essentially controlled by the properties of the contacting surfaces (metallurgy, surface roughness, hardness) and the nature of the SF lubricating film that is formed during the gait cycle. The relevant lubricating properties are the film thickness and chemical composition; both are determined by the prevalent film formation mechanism occurring during the gait cycle. At present there are two broad schools of thought concerning these mechanisms: boundary lubrication [**4**, **5**], where film formation is determined by the chemistry at the surface; and fluid film lubrication, which obeys classical elastohydrodynamic theory [**6**].

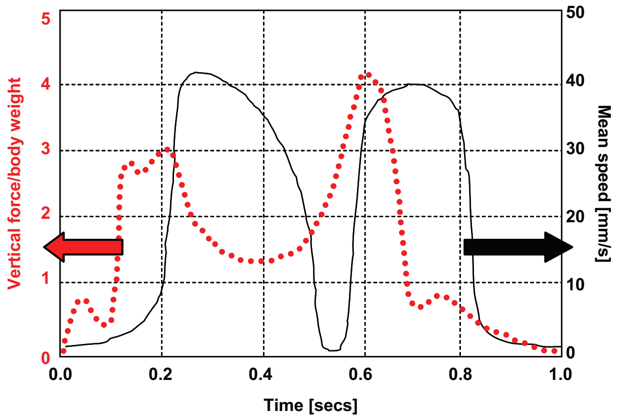

**Fig. 1** Representation of standard hip gait cycle

The kinematics of hip joint articulation is complex as the contact undergoes transient loading and speeds over the gait cycle [**6**]. A simplified example is shown in Fig. 1. The joint will typically experience two loading peaks per cycle (~1 Hz), with linear speeds of 2–50 mm/s. The fluctuating speed and load conditions will probably result in the predominant film formation mechanism (boundary to fluid film) varying over the gait cycle. Central elastohydrodynamic (EHL) film thickness is typically predicted to be in the range of 30–70 nm over the gait cycle [**6**] for SF.

SF is a viscoelastic shear-thinning fluid that is composed of a complex mixture of large and surface-active molecules including proteins, hyaluronic acid, and phospholipids [**7**, **8**]. SF is non-Newtonian, but at physiological shear rates the effective viscosity (for film thickness calculations) is usually assumed to be constant (for example 0.0025 Pa s [**6**]). The chemical and physical properties of SF are affected by disease (osteoarthritis and rheumatoid arthritis) and trauma. This can result in a significant decrease in the viscosity and changes in the protein content and pH [**8**, **9**].

It is widely recognized that proteins play an important role the lubrication process. Healthy SF contains albumin and globulin at concentrations of 0.7–1.8 g/dl and 0.05–0.29 g/dl, respectively [**9**]. In periprosthetic SF the protein content increases [**8**]. However, it is more common to use bovine serum (BS), typically diluted with water to a concentration of 25 per cent w/w, as model SF in hip simulator tests [**10–12**]. Wear studies, in both hip simulators and pin-on-disc tests, have shown a correlation between the protein content and wear [**10**, **11**]. Examination of metal implant surfaces from both *in vitro* and *in vivo* function has shown that proteins are deposited on the surfaces [**12**, **13**] and are implicated in boundary film formation [**10**].

Recent work [**14**] in the authors' research programme has shown that BS and protein solutions form abnormally thick films at low sliding speeds. Such behaviour has important implications for surface protection at low speed, start-up, or at stance. The origins of this film behaviour and the role of the SF proteins are the topic of the present study.

This study reports film thickness measurements for a range of BS and proteins solutions. The experimental method differs from the earlier publication [**14**] as a commercial cobalt–chromium (CoCrMo) metal femoral head was used as one of the rubbing surfaces. This change provides a significant improvement of the authors' laboratory simulation of the problem.

## 2 RESEARCH PROGRAMME

The research programme was designed to study the fundamental mechanisms of SF lubrication, and in particular the role of proteins in film formation. BS and protein solutions (albumin and $\gamma$-globulin) were used as the test fluids. The research programme included the following:

(a) film thickness measurements and in-contact imaging to study lubricating film formation in a sliding contact;
(b) optical examination of the metal surface before and after film thickness testing;
(c) measurement of surface roughness changes using optical profilometry;
(d) Fourier transform infrared (FTIR) spectroscopic analysis of surface deposits close to the rubbed area on the femoral head.

An optical interferometric method was used to measure film thickness for a commercial CoCrMo alloy head rubbing against a glass disc. All measurements were made at 37 °C.

The protein and BS test fluids were made using deionized water (DIW). For the present study it was decided not to simulate physiological pH as the ionic salts used in buffer solutions are expected to contribute to surface film formation, thus obscuring the protein lubricating mechanisms. Protein solutions were made with DIW; however, the poor solubility of $\gamma$-globulin in DIW meant that only a limited concentration range could be studied. This approach will be extended in later work where film formation under different pH conditions and in the presence of ionic salts (physiological and buffer) will be examined.

**Table 1** Measured viscosities, pH levels, and protein concentrations for test solutions

| Composition | Viscosity (Pa s) at $10^3$ $s^{-1}$ | pH at 19 °C | Total protein concentration (g/dl) |
|---|---|---|---|
| 100 % w/w BS | 0.026 | 7.60 | 6.45 |
| 50 % w/w BS | 0.022 | 7.54 | 3.23 |
| 25 % w/w BS | 0.021 | 7.34 | 1.62 |
| Albumin solution | – | 6.91 | 1 |
| γ-Globulin solution | – | 7.28 | 0.18 |
| Albumin/γ-globulin solution | – | 7.18 | 1.18 |

## 3 EXPERIMENTAL PROCEDURE

### 3.1 Test fluids

Two groups of test fluids were used.

1. Bovine serum: bovine calf serum (BS; Sigma Aldrich 12133C, protein concentration 5.8–7.1 g/dl). BS concentrations of 100, 50, and 25 per cent w/w were tested.
2. Protein solutions: single protein solutions were prepared containing albumin and γ-globulin at a concentration within the range for healthy SF [**9**].

All solutions were prepared with deionized water (Sigma-Aldrich S-37531-356) to the appropriate w/w concentration. The solutions were stored in a fridge (5°C) immediately after preparation and used within 1 week; consequently it was not necessary to use antibacterial agents. Test solution properties are summarized in Table 1; measured viscosity, pH level, and protein concentrations are given for all test lubricants. Approximate protein concentrations are given for BS solutions as quoted by the supplier.

The test fluid rheology was measured using a cone-on-plate rheometer (AR2000; TA Instruments) with a hard-anodized aluminium peltier cone. Steady-state flow tests were carried out where shear stress was measured as a function of shear rate. The experimental protocol used was similar to that followed by Oates *et al.* [**15**]. The fluids were presheared at 60 $s^{-1}$ for 5 min to eliminate any previous shear history. The shear rate was then increased from 0.002 to 1000 $s^{-1}$ and viscosity measurements taken over the range. Each measurement was repeated twice. The effective viscosity measured at $10^3$ $s^{-1}$ was used to calculate film thickness.

### 3.2 Film thickness measurements

Lubricating film thickness at the centre of the contact was measured in a ball-on-disc device (PCS Instruments) using thin-film optical interferometry [**16**]. A diagram of the test device is shown in Fig. 2(a). The underside of the glass disc was coated with a thin (10 nm) chromium coating overlaid by a thicker silica layer (500 nm). The coatings provide the necessary reflection conditions for the interferometric method. The technique measures central film thickness in the range 1–1000 nm with a resolution of ±1 nm. A personal computer controlled the temperature, load, and sliding speeds. Film thickness test conditions are summarized in Table 2.

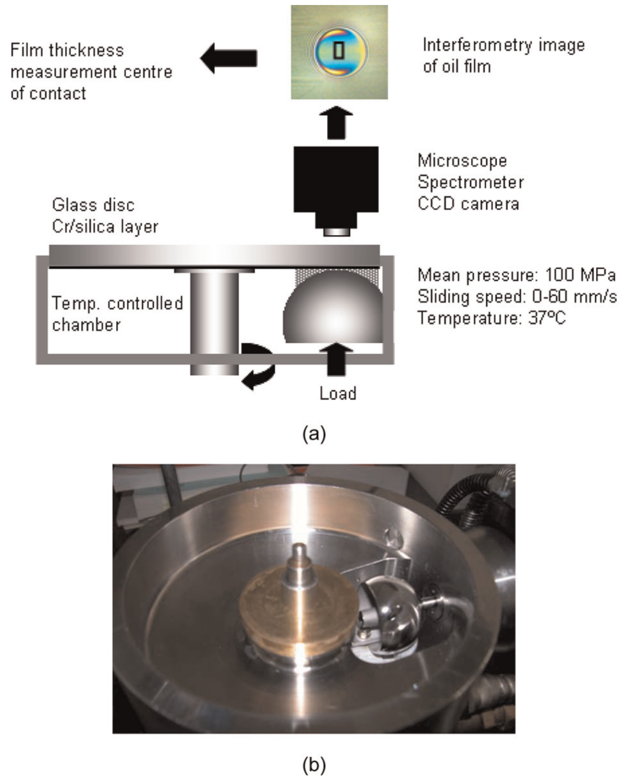

**Fig. 2** Film thickness measurement device: (a) schematic diagram; (b) photograph of femoral head mounted in test device

A resurfacing femoral head (CoCrMo alloy, 38 mm diameter, non-heat-treated) was used as the stationary counterface. This was held in the test device and loaded against the underside of the rotating glass disc, which was driven by an electric motor. The head was held in a supporting mount (see Fig. 2(b)) and could be rotated so that different parts of the head could be tested. A new sample area was used for each test.

The glass disc and femoral head were cleaned in 1 % w/w didodecyl sulfonate solution in an ultrasonic bath, rinsed repeatedly in distilled water, and then washed in acetone before air-drying and assembly. The test device was preheated to 37°C and the entire assembly was allowed to reach

**Table 2** Film thickness test conditions

| Parameter | Value |
|---|---|
| Test conditions | |
| Contact pressure | 100 MPa |
| Speed range | 2–60 mm/s |
| Temperature | 37°C |
| Specimens | |
| Upper window | Glass disc |
| Lower specimen | CoCrMo resurfacing femoral head, 38 mm diameter |

thermal equilibrium before the test was started. Test fluids were pumped into the contact zone to avoid contamination with hydrocarbons from the shaft bearings and seals. The lubricant reservoir was held in a burette outside the test chamber. Silicone tubing was used to feed the fluid into the exit region, which ensured that an extensive meniscus was maintained around the contact. It also provided a thick fluid film layer in the exit so that the effects of evaporation were minimized as the disc rotated. The silicone tubing was held within the heated test chamber to ensure that the test fluid was at the correct temperature. A thermocouple positioned at the exit of the silicone tubing was used to monitor the fluid feed temperature.

Film thickness was measured as a function of mean speed over the range of 2–60 mm/s, which corresponds to the range of linear speeds occurring during hip joint articulation. Film thickness readings were taken periodically over the entire speed range. Tests were carried out by slowly increasing the disc speed in steps from 2 to 60 mm/s (results labelled 'UP1') then decreasing (results labelled 'DOWN1'). The speed increase/decrease curves were then repeated ('UP2'/'DOWN2'). At the end of each test, the residual film thickness was measured under static loading (5 N); this provided an indication of the formation of deposited or adsorbed layers at the surface. These layers are able to maintain surface-separation under loading.

The load was held constant during the test at 5 N; this is not representative of a real hip joint which experiences transient conditions during the gait cycle. However it does provide a measurement of the film thickness under the most severe loading (i.e. heel strike) and presumably high wear condition.

The BS results were compared with theoretical values plotted in the graphs. The film predictions were calculated from the isoviscous film formula from Hooke [**17**] as follows

$$h = 4.18\frac{(U\eta)^{0.6}R'^{0.67}}{W^{0.13}E'^{0.47}} \tag{1}$$

where $U$ is the entrainment speed; $\eta$ is the viscosity of the lubricant; $R'$ is the reduced radius, which for this case is the radius of the femoral head; $W$ is the applied load; and $E'$ is the reduced Young's modulus, which is given by the formula

$$\frac{2}{E'} = \frac{1-\nu_1^2}{E_1} + \frac{1-\nu_2^2}{E_2} \tag{2}$$

where $\nu$ is the Poisson's ratio and $E$ is the Young's modulus for the femoral head and the glass disc denoted as 1 and 2, respectively.

In some tests a charge-coupled device (CCD) camera was mounted on the microscope and images captured from the contact zone during the test. Images were taken at the start, at different speeds, and at the end of the test sequence.

### 3.3 Examination of worn surfaces

The femoral head surface was examined before and after testing using an optical microscope and optical interferometric surface profilometer (Wyko NT1100). The femoral head was rinsed with DIW to remove the bulk fluid prior to examination. In a limited number of cases an FTIR spectrometer (Perkin Elmer Spectrum One) coupled to an FTIR microscope (Perkin Elmer Multiscope) was used to analyse surface deposits (100 $\mu$m sample area) near the wear scar on the femoral head. IR reflection–adsorption spectroscopy (IRRAS) provides information on the chemical composition and distribution of lubricant films on metal surfaces [**18**]. FTIR spectroscopy has been used extensively to characterize the composition and molecular conformation of protein films [**19**].

It was also interesting to compare the laboratory tests with those from explanted joints. These were received from the London Implant Retrieval Centre (LIRC). The LIRC was established in 2008 by one of the authors (A.H.) to analysis causes of failure in explanted MOM hips. As part of that work, wear and surface roughness measurements are made from the explanted metal components [**20**].

## 4 RESULTS

### 4.1 Film thickness measurements

Typical film thickness measurements for BS concentrations of 25, 50, and 100 per cent w/w are presented in Figs 3 to 5. Central film thickness is plotted against mean speed and four different curves are shown for each fluid. Film thickness results for the protein solutions are given in Figs 6

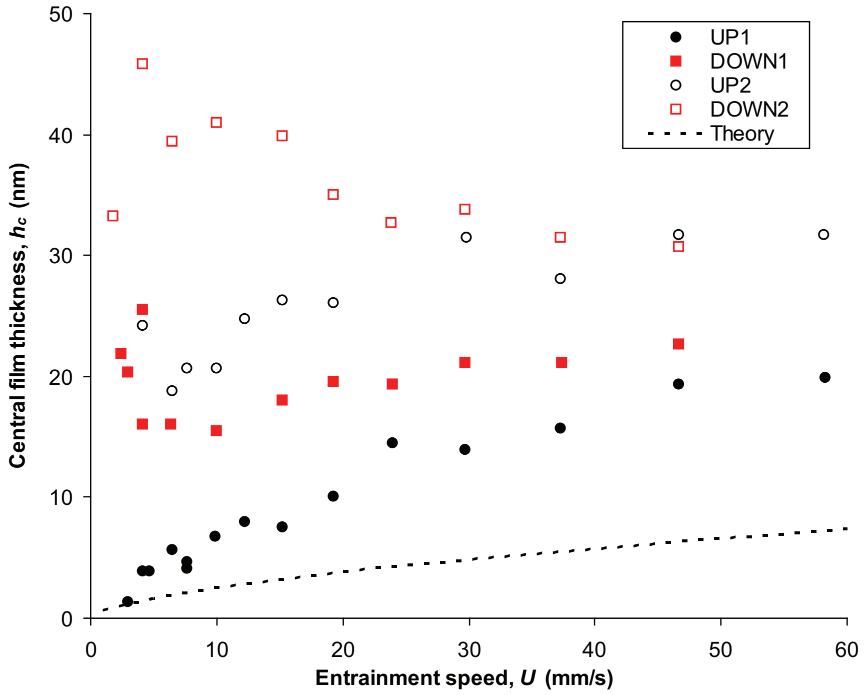

**Fig. 3** Film thickness results for BS concentration of 25 % w/w, with calculated result [**17**] plotted as a dotted line

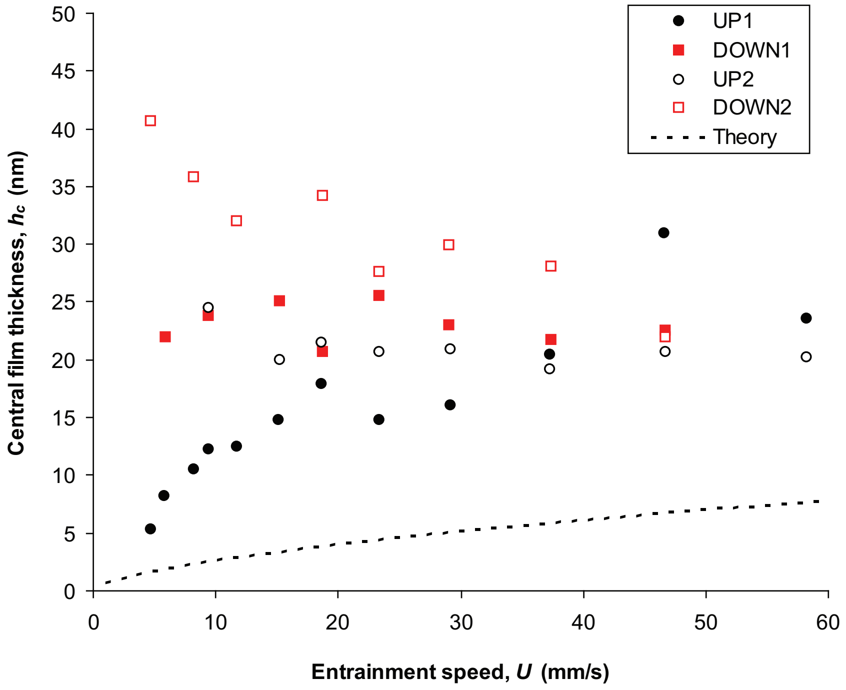

**Fig. 4** Film thickness results for BS concentration of 50 % w/w, with calculated result [**17**] plotted as a dotted line and 7. At the end of the tests the residual film was measured under static loaded conditions. These results are summarized in Table 3.

The film results for the BS solutions are complex and time dependent. Initial speed curves (UP1) obeyed classical EHL rules and increased with speed. The predicted film thickness is also plotted for each of the BS concentrations. However subsequent curves showed very different behaviour. Because of the complexity of the results only one

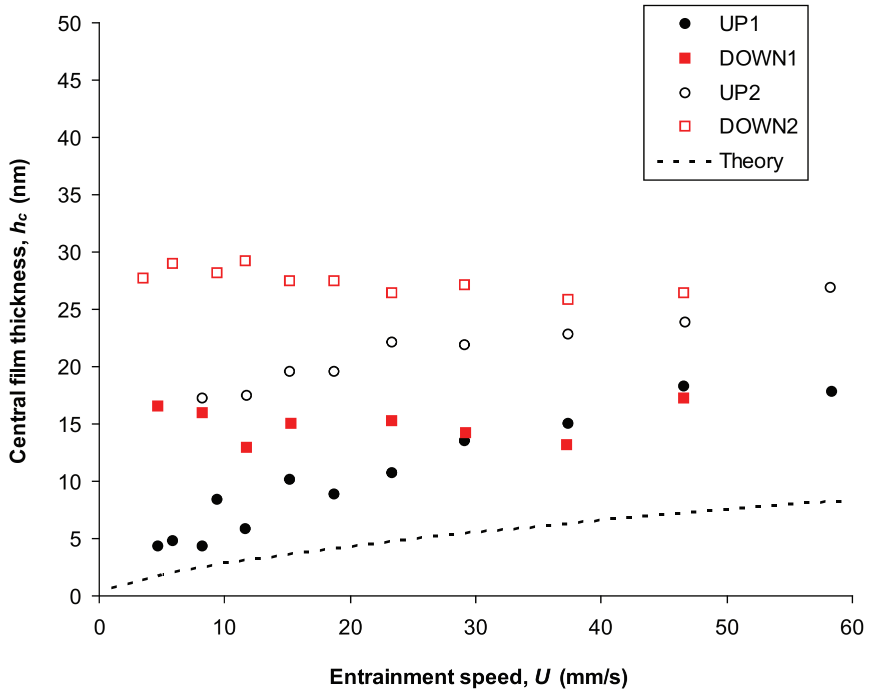

**Fig. 5** Film thickness results for BS concentration of 100 % w/w, with calculated result [**17**] plotted as a dotted line test is shown for each fluid. However the tests were repeated at least twice and the pattern was the same each time; results representative of this behaviour are shown.

The results in Fig. 3 are typical of the speed–time behaviour observed with BS solutions. In the initial curve (UP1) film thickness increases with speed to a maximum of ~20 nm at 60 mm/s. Decreasing speed gives a very different result; the film thickness remains fairly constant (15–19 nm) down to 5 mm/s when it starts to increase again. Subsequent speed increase/decrease curves are similar except the overall film thickness is now in the range 20–50 nm. At the end of the test, the residual film (film thickness measured at 0 mm/s, 5 N load) is typically in the range 20–40 nm.

The results for BS concentrations of 50 and 100 per cent w/w are shown in Figs 4 and 5. Again very similar behaviour is seen; the initial film thickness increases with speed followed by growth of the film with increasing time or rubbing. These results can be summarized as follows.

1. The initial film thickness increased with speed to a maximum of 16–20 nm at ~60 mm/s.
2. The overall film thickness increases with each subsequent speed curve. The final results are typically in the range 25–50 nm. The thicker films are usually seen for the lower BS concentration. The thickest films are usually recorded at the lowest speeds.
3. A residual surface film is deposited within the contact region. This is in the range of 20–50 nm.

Figure 6 shows representative results for the single protein solutions: albumin (1 g/dl) and $\gamma$-globulin (0.18 g/dl). The results for albumin show the same characteristic curves as the BS solutions. The final curve is in the range 40–60 nm. The $\gamma$-globulin solution, despite the much lower concentration, gives slightly thicker films with a final curve of 65–80 nm.

The mixed protein results are shown in Fig. 7. The pattern of film formation is very similar to the albumin test. A final film in the range of 50–80 nm is obtained. The residual film thicknesses measured at the end of the tests are summarized in Table 3. The overall range for these measurements is 20–60 nm.

## 4.2 In-contact imaging

In-contact images (field of view 1.2 mm×0.96 mm) are shown in Fig. 8 for 25 per cent w/w BS at different speeds (under 5 N load) as follows: 0 mm/s (start), 5 mm/s, and 20 mm/s.

Figure 8(a) shows the loaded contact at the start of the test. The blue region is the Hertzian contact zone (blue coloration is due to the silica spacer layer). Outside the Hertzian region the femoral head surface appears corrugated – this texturing is

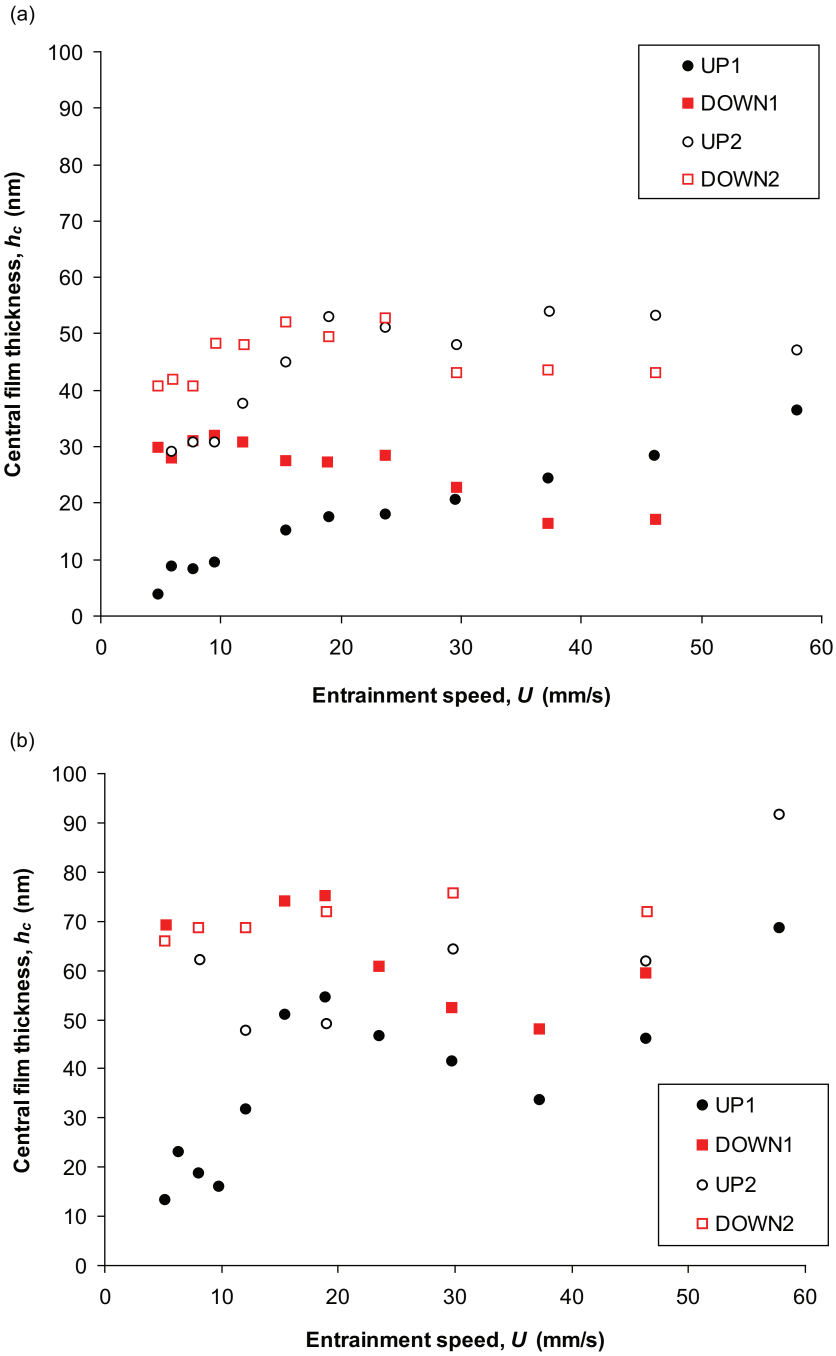

**Fig. 6** Film thickness results for single proteins: (a) albumin, 1 g/dl; (b) $\gamma$-globulin, 0.18 g/dl discussed in section 4.4. As the disc starts to rotate a film builds up rapidly in the contact zone; this is seen in localized regions (Fig. 8(b)) particularly at low speeds. At higher speed (Fig. 8(c)) this film tends to break down.

### 4.3 Examination of the femoral head contact region

Images of the femoral head contact region without the glass disc present were also recorded at the start and end of the test (after cleaning). An example is shown in Fig. 9; these images were taken before (Fig. 9(a)) and after (Fig. 9(b)) the same test with 25 per cent w/w BS.

Figure 9(a) shows the surface texture of the new femoral head; it is characterized by 'block' features typical of as-cast CoCrMo alloys. Examination of the wear surface at the end of the test showed loss of the carbides and the formation of a wear scar. These block structures were lost rapidly at the start of the test and this was accompanied by the

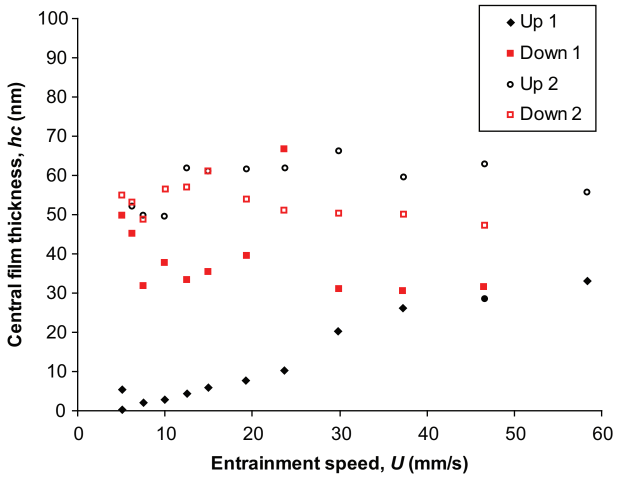

**Fig. 7** Film thickness results for mixed albumin/γ-globulin protein solution

**Table 3** Summary of residual film thickness measurements (loaded, static contact at end of test)

| Lubricant | Residual film thickness (nm ± range) |
|---|---|
| BS, 25 % w/w | 35.75 ± 2.5 |
| BS, 50 % w/w | 19.4 ± 3.5 |
| BS, 100 % w/w | 34 ± 0.00 |
| Albumin, 1 g/dl | 52.95 ± 24.05 |
| γ-Globulin, 0.18 g/dl | 29.55 ± 14.00 |
| Albumin/γ-globulin, 2:1 | 60.00 ± 20.00 |

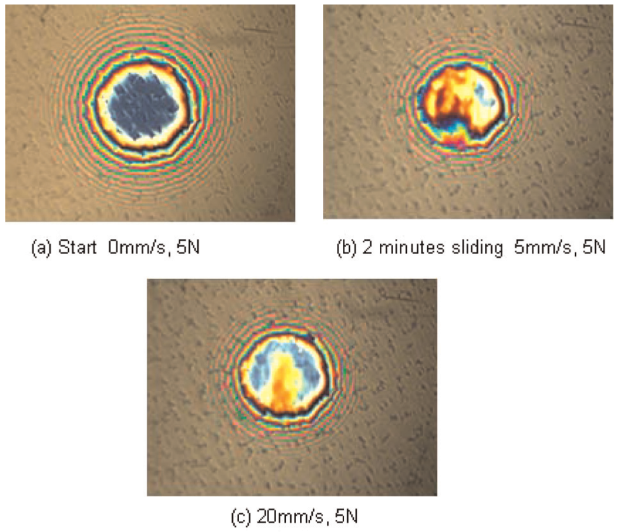

**Fig. 8** Images from the contact zone during sliding at different speeds for 25 % w/w BS (field of view 1.2 mm×0.96 mm)

formation of a wear scar, which can be seen in Fig. 9(b). This was an extremely surprising result as the head was rubbing against a glass disc covered by a chromium/silica layer. Scratches were also observed in the direction of sliding, these were

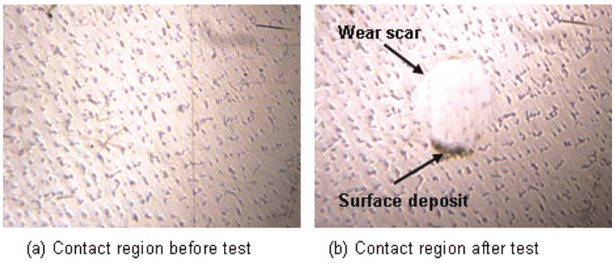

**Fig. 9** Optical images from the femoral head before and after testing (field of view 1.2 mm × 0.96 mm)

attributed to third body wear [**21**] due to abrasion by carbide particles embedding in the surface of the glass disc. No damage was observed to the silica coating during the test, which lasted for less than 15 min.

Figure 10 shows line profiles taken from inside and adjacent to the wear scar. Loss of the surface block structures occurs within the contact zone. The block structures on the unused surface are typically 80–100 nm high with typical surface dimensions of 40 $\mu$m×15 $\mu$m.

Optical examination of the femoral head at the end of the test showed deposited surface films in and around the contact patch. These deposits often remained after light rinsing and it was possible to analyse their chemical composition using IRRAS. Figure 11 shows a spectrum taken from the surface deposits on the inlet side of the wear scar (similar to those shown in Fig. 9(b)). The test fluid was 25 per cent w/w BS solution. The spectrum shows intense absorbance bands at ~1650 and 1535 $cm^{-1}$ that are characteristic of protein amide I ($\nu_{C=O}$) and II ($\nu_{C-N}$, $\delta_{N-H}$) band vibrations. The IR reflection spectra confirm the deposits are proteins; however, the analysis can also provide insights as to the molecular conformation. The amide I/amide II absorbance ratio and the frequency of the amide 1 band have been used as indicators of protein conformation [**19**]. The relatively low frequency of the amide I band suggests the 'presence of packed hydrophobic helical domains' [**19**]. The use of IR reflection spectra to examine the conformation and composition of protein boundary films will be explored in a later paper.

## 5 DISCUSSION

### 5.1 Film formation mechanisms

The film thickness results demonstrated complex, time-dependent behaviour of the BS and protein solutions. At the start film thickness increased with

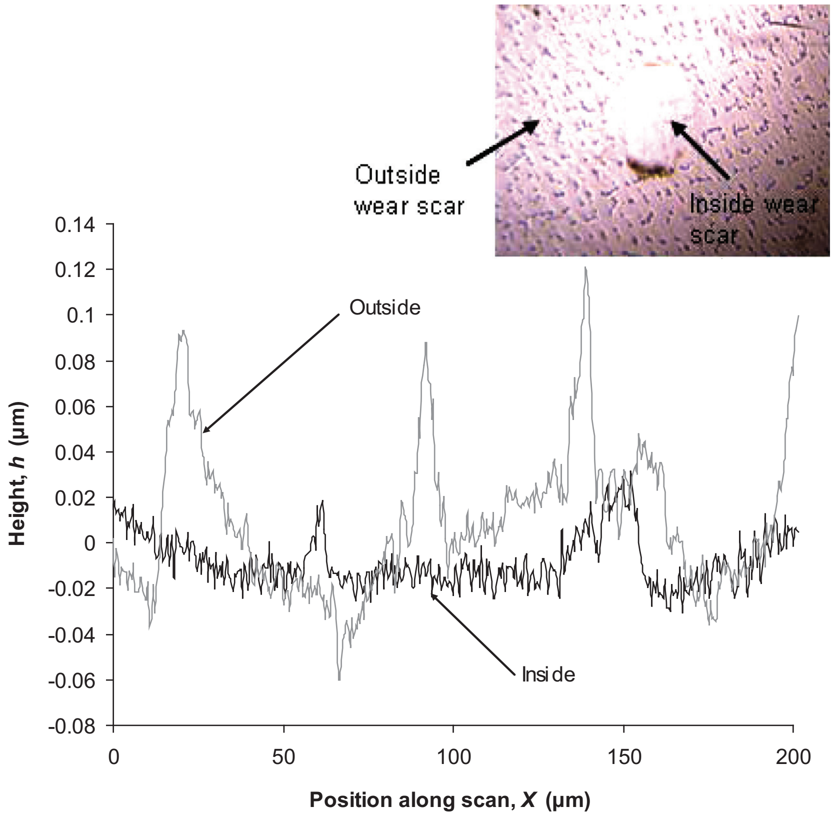

**Fig. 10** Interferometric roughness images of femoral head surface texture outside and inside the wear scar

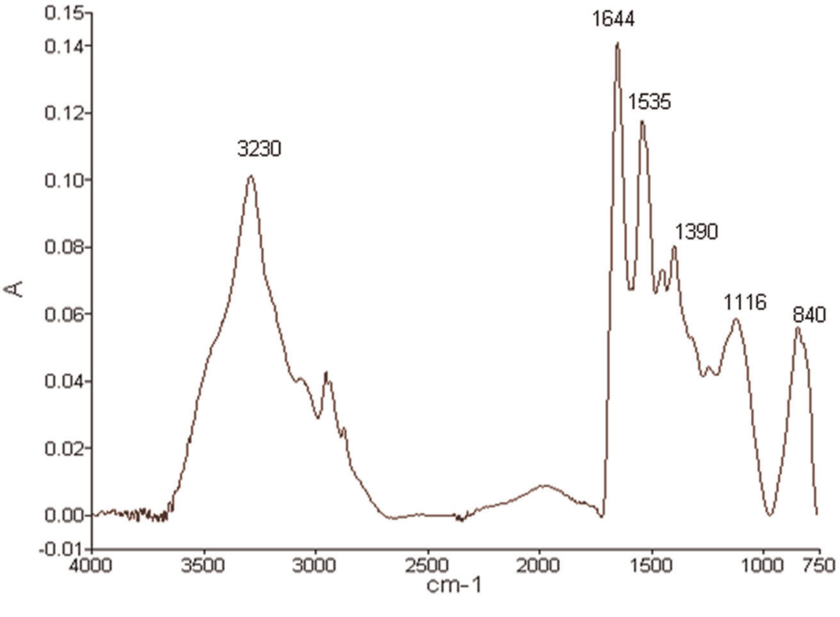

**Fig. 11** FTIR micro-reflection spectra from the wear scar deposits, BS solution speed as expected from an EHL model [**17**]. Subsequent speed curves showed significant deviation from the fluid film prediction. At present EHL theories (to predict film thickness) are based on simple continuum fluids and do not account for the type of behaviour observed here. The results suggest that protein aggregation and deposition cause complex film formation mechanisms. Film thickness tended to increase particularly at low speeds; this often resulted in an almost speed-independent film over the speed range. The same film-building behaviour was obtained with all test fluids, suggesting it is characteristic of protein-containing solutions. Film formation at lower speeds is often associated with the formation of a phase boundary in the inlet region. Clearly the film thickness distribution shown in Figs 8(b) and (c) does not resemble a simple fluid film (see Fig. 2(a) for a classical elastohydrodynamic film pattern).

By analysing the film thickness–speed behaviour it is possible to infer properties of the films and gain an insight into the formation mechanisms. After completion of the sliding tests under static loaded conditions ($U = 0$, $W = 5$ N), the residual film between the surfaces was measured. Overall the residual films were in the range of 20–60 nm, although the thicker films were often observed to decrease with prolonged loading. Earlier work [**14**] also showed deposition of solid films in the contact; although these were thinner in the range of 10–20 nm. However these measurements were taken after two speed sweeps, while in the current tests four speed sweeps were used. The nature of the residual film formed in these tests was also different. In the earlier work the film appeared to be 'solid' and did not decrease significantly with time under load. In the current study the authors observed that for some measurements the thickness did drop slightly with time, suggesting a component of the film was high-viscosity material rather than a solid adherent layer. This effect will be studied in more detail in a subsequent paper. This observation supports the earlier conclusion that the film is biphasic [**14**], comprising a thin adherent film overlaid by a much thicker, high-viscosity, loosely-bound component. The thin films appeared to be securely bound to the surfaces and were not removed by repeated loading. These are likely to be composed of protein molecules adsorbed at the metal or glass surfaces to form multi-molecular layers. The adsorption of protein molecules from flowing solutions at interfaces has been the subject of considerable research because it is of importance in many industrial areas such as the food industry [**22**, **23**]. A number of different models have been proposed for the initial monolayer formation, including reversible/irreversible Langmuir adsorbance, protein exchange reactions, and molecular conformational changes (see Introduction to reference [**22**]). The formation of a precursor monolayer might then be followed by multilayer formation [**22**, **24**]. Proteins are also known to aggregate in solution [**22**], under shear [**22**], at surfaces [**21**], and after heat treatment [**22**, **23**], and these structures may be involved in the multilayer formation. The agglomerate can be large; for example, Santos *et al.* [**22**] reported hydrodynamic radii of 3 nm for native species and 110–160 nm for aggregates.

The present results indicate that a 'high-viscosity' layer augmented the residual film. At low speeds BS often forms much thicker films than expected, although there is considerable fluctuation in the results. This behaviour appears to be associated with the formation of a new phase boundary in the inlet, which is indicated by a change in refractive index (step change in interference contours). The new phase is attributed to a local concentration of protein molecules forming a gel-like deposit. This material passes through the contact in a non-uniform manner causing the film thickness to fluctuate and is responsible for the scatter in the results. The new phase builds up locally in the inlet at slow speeds to form a reservoir of high-viscosity material. As the speed increases this tends to break down and as a result the film thickness often drops at higher speeds. The local concentration and deposition of protein molecules is thought to be due to shear-induced aggregation in the inlet region [**22**, **25**]. The residual film thickness will include this 'inlet agglomeration' component (possibly as an entrapment) as the measurements were made at the end of the slow speed cycle. It is unclear why this mechanism breaks down at higher speeds; possibly the protein agglomerations are swept around the contact and do not contribute to film formation. However, as the MOM head/cup contact is highly conformal with a semi-contact width of ~5 mm (50 mm diameter head, 100 $\mu$m clearance, 3500 N), in this case it is unlikely that protein agglomerations can escape entrainment quite so easily.

The main conclusion from this work is that the lubrication mechanism of protein solutions in sliding contacts is complex and not described by simple fluid considerations. Film formation is dominated by surface deposition and shear-flow aggregation of protein molecules. A solid film of 10–20 nm is formed initially which the authors believe is due to adsorbance of protein molecules at the rubbing surface; this is augmented by a thicker, hydrodynamically generated film of high-viscosity material. Protein molecules are aggregated in the inlet shear field and come out of solution to form gel-like deposits in the inlet; this material adheres to the metal surface and periodically passes through the contact forming a much thicker hydrodynamic film.

The formation of 'gel-like' deposits has been reported in earlier studies [**1**, **26**]. In their study of the wear of ultrahigh-molecular-weight polyethylene, Wang *et al.* [**1**] suggested that these deposited films provided surface protection and soluble proteins did not contribute to boundary lubrication. Lu and McKellop [**26**] also concluded that protein molecules denatured by frictional heating in the contact zone formed the surface deposits. It is possible that thermal effects within the contact zone, in particular towards the exit region and pressure constriction, aid protein surface adhesion. However, post-test rinsing and examination of the femoral head showed greater protein adhesion within the inlet region, so it is unlikely that a large temperature increase will occur here. This suggests that shear flow dominates protein aggregation and deposition within the inlet region. The question of protein solubility is interesting; in tests not reported in this paper, thicker films were obtained with unfiltered protein solutions (compared with filtered) that supports the idea of surface deposition of insoluble proteins. Wimmer *et al.* [**12**] have previously reported the presence of deposited proteins on the surface of explanted joints and the current findings are in accord with this. Clearly protein solubility, including the effect of pH and ionic strength, and surface adherence are important issues and these will be studied in later papers. One interesting observation from this work is that protein deposits are formed in the inlet of the contact in the low pressure region. Other papers have suggested that denaturing of proteins in the Hertzian contact, due to high pressures or temperatures, is responsible for the formation; however, it is concluded here that flow aggregation is the dominant mechanism.

### 5.2 Surface topography of wear scars

In addition to the protein deposition other changes occurred in the contact zone during testing. Surface block structures were rapidly removed at the start of sliding and a wear scar developed, often accompanied by the formation of surface scratches in the direction of sliding. The CoCrMo test specimen was a commercial as-cast resurfacing component. The metallurgy of the CoCrMo alloy and the effect of casting methods on wear have been studied extensively [**27**, **28**]. The block structures observed on the surface of the femoral head are usually described as ‘carbides’ [**27**] and have high molybdenum, chromium, and carbon content [**27**, **28**]. The optical surface profilometry measurement showed the features had ‘block’ morphology [**27**] and were approximately 80–100 nm high with upper surface dimensions of approximately 50 $\mu$m×20 $\mu$m. The very rapid loss of these carbides, usually within a few minutes of the start of sliding, was unexpected; particularly as the silica coating appeared undamaged.

It is interesting to compare these observations from laboratory tests with those from LIRC explanted joints. An example is shown in Fig. 12, which compares optical profilometer images of worn and unworn areas on an acetabular cup (same manufacturer as the present test specimen). The block pattern features are clearly still present outside the wear scar (Fig. 12(b)); these have similar dimensions to our test specimen. Within the wear scar these features are lost and scratches are observed which are typically 3 $\mu$m wide and 30–80 nm deep (Fig. 12(c)); similar to those found in the laboratory tests.

### 5.3 Caveat

Although these results appear to offer insights into protein lubrication mechanism in artificial hip joints there are caveats. A number of issues have not been investigated in this paper and these should be addressed before the relevance of this mechanism for *in vivo* joint lubrication can be assessed. These issues include contact conformity and clearance and the effect of surface wetting on film formation. The test configuration used in the current work utilizes a stationary head loaded against a moving glass disc. In reality neither surface is stationary, so that inlet build-up is unlikely to occur over a significant time scale. However similar entrainment speeds and contact pressures are experienced in a functioning artificial hip joint. Therefore it is possible that this mechanism does take place and is responsible for localized concentration of proteins that pass into the contact zone. In the high-pressure Hertzian zone the proteins are deposited as an adherent surface layer.

The solutions used here do not replicate *in vivo* lubricants, although 25 per cent w/w BS in DIW is the standard in hip simulator testing. Thus the paper may be considered to be a simulation of *in vitro* rather than *in vivo* use. In future work the effects of pH, ionic salt content, and other SF components (dipalmitoylphosphatidylcholine, hyaluronic acid) on film formation will be examined.

## 6 CONCLUSIONS

The paper has studied film formation by protein and BS solutions in a sliding test device. Film thickness measurements were made over a speed range of 2–60 mm/s using a CoCrMo femoral head as a stationary counterface. The following conclusions may be drawn.

1. Protein-containing solutions demonstrate complex time-dependent film thickness behaviour

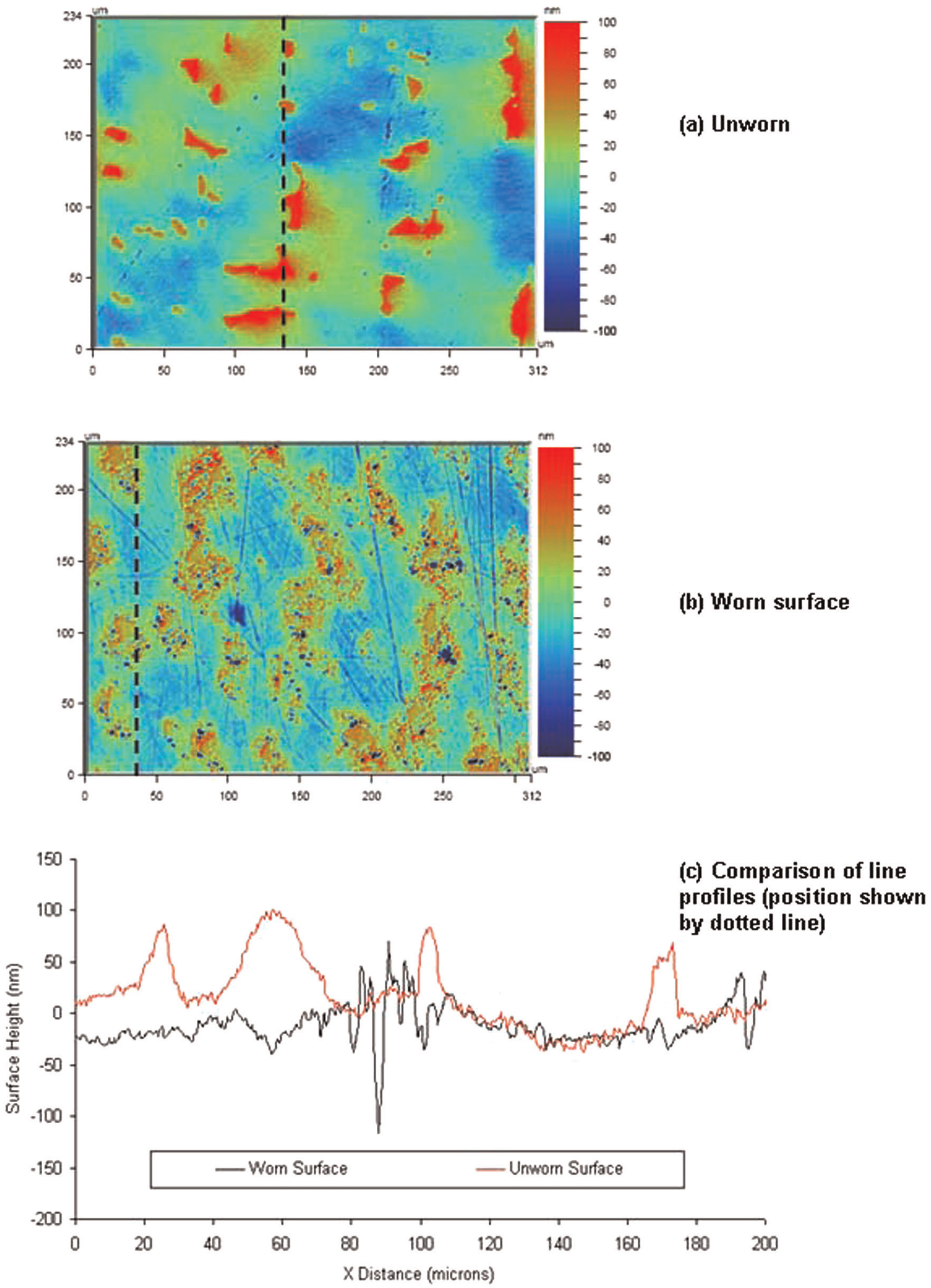

**Fig. 12** Optical images and line profiles from explant wear surface (position of line profile measurement indicated by the dotted line)

that is not characteristic of a simple Newtonian fluid.
2. Protein-containing solutions form abnormally thick (20–50 nm) films in the low speed region.
3. These films are composed of a thin (~1020 nm) solid adsorbed layer, augmented by a thicker hydrodynamic-generated film.
4. The low-speed film behaviour is thought to be due to the development of a reservoir of high-viscosity material in the inlet which periodically passes through the contact, forming much thicker films.
5. The inlet material is composed of protein molecules agglomerated by shear flow.
6. CoCrMo block structures on the surface are rapidly removed by rubbing and appear to contribute to further damage by abrasion.

## ACKNOWLEDGEMENTS

The authors wish to thank the EPSRC for funding this research (EP/E028721/1) and Dr A. Mavraki for carrying out the rheology measurements.
